# The Effect of Serotonin 5‐HT_2C_ Receptor Modulation on Binge Drinking and Alcohol‐Seeking in Female Mice

**DOI:** 10.1111/adb.70099

**Published:** 2025-11-10

**Authors:** Roberta G. Anversa, Linh Tran, Andrew A. Bolinger, Jia Zhou, Kathryn A. Cunningham, Andrew J. Lawrence, Erin J. Campbell

**Affiliations:** ^1^ Florey Institute of Neuroscience and Mental Health, Melbourne Brain Centre The University of Melbourne Parkville VIC Australia; ^2^ School of Biomedical Sciences and Pharmacy, Faculty of Health and Medicine University of Newcastle Callaghan NSW Australia; ^3^ Brain Neuromodulation Research Program Hunter Medical Research Institute New Lambton Heights NSW Australia; ^4^ Center for Addiction Sciences and Therapeutics, and Department of Pharmacology and Toxicology University of Texas Medical Branch Galveston Texas USA

**Keywords:** 5‐HT_2C_R positive allosteric modulators, alcohol, alcohol use disorder, binge drinking, lorcaserin, serotonin

## Abstract

Alcohol misuse remains a leading cause of preventable death worldwide, prompting research into novel pharmacotherapies for alcohol use disorder (AUD). This study investigated the therapeutic potential of full agonism or positive allosteric modulation of the serotonin 2C receptor (5‐HT_2C_R) in addressing alcohol binge drinking and seeking behaviours in mice. Using a drinking‐in‐the‐dark paradigm and a context‐induced reinstatement model following punishment‐imposed abstinence, we assessed the acute effects of 5‐HT_2C_R ligands lorcaserin, CYD‐1‐79, VA012 and CTW0415 on alcohol intake and seeking behaviours in mice. Results showed that while lorcaserin effectively reduced both alcohol consumption and seeking behaviours, the 5‐HT_2C_R positive allosteric modulators (PAMs) did not significantly alter these behaviours over the range of doses examined. These findings suggest that 5‐HT_2C_R PAMs, at the tested doses, may lack intrinsic efficacy in modulating alcohol use. However, our lorcaserin data demonstrate that targeting 5‐HT_2C_R remains a valid approach to reduce behaviours associated with AUD.

## Graphical abstract

Alcohol misuse remains a leading cause of preventable death worldwide, prompting research into novel pharmacotherapies for alcohol use disorder. Here we showed that lorcaserin, a potent serotonin 2C receptor (5‐HT_2C_R) agonist, effectively reduced alcohol consumption and seeking behaviours in mice. Despite concerns over potential off‐target effects with long‐term lorcaserin use, our data demonstrate that targeting 5‐HT_2C_R remains a valid approach to reduce behaviours associated with alcohol use disorder.

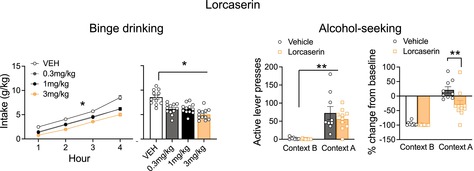

## 1 Introduction

Alcohol misuse is one of the leading causes of preventable death throughout the world [1]. Although the US Food and Drug Administration (FDA) has approved pharmacotherapies to treat alcohol use disorder (AUD), these often have adverse side effects and low compliance rates [2, 3]. Thus, more recently, the AUD field has begun to unravel the specific neurochemical mechanisms of this disorder to inform targeted medication development and reduce side effects [4]. One such system that has been implicated in AUD is the serotonin (5‐hydroxytryptamine, 5‐HT) system.

5‐HT neurons are primarily located in the raphe nuclei of the mid‐ and hindbrain [5]. These neurons project widely throughout the brain, including to several reward‐related brain regions such as the striatum, amygdala and hippocampus [6]. There are 14 known subtypes of 5‐HT receptors, and one with relevance for AUD is the 5‐HT_2C_ receptor (5‐HT_2C_R). In rodent pharmacological studies, administration of a 5‐HT_2C_R agonist decreased alcohol consumption, while a 5‐HT_2C_R antagonist increased alcohol consumption [7]. More recently, chemogenetic inhibition of subcortical 5‐HT_2C_Rs increased alcohol self‐administration, and chemogenetic activation of subcortical 5‐HT_2C_Rs reduced alcohol consumption [8, 9]. The 5‐HT_2C_R is also implicated in human alcohol use. We have recently shown that lorcaserin, a potent 5‐HT_2C_R agonist initially approved for weight loss treatment, reduced craving for alcohol in treatment‐seeking individuals with AUD [10]. Unfortunately, lorcaserin was subsequently withdrawn from the market due to concerns over potential off‐target effects with long‐term use [11]. This prompted us to investigate new 5‐HT_2C_R ligands that could retain efficacy while minimising long‐term off‐target effects.

Lorcaserin binds to the orthosteric 5‐HT_2C_R site that hosts endogenous 5‐HT mechanisms, which are complex and involve several downstream signalling pathways [12]. Recent medicinal chemistry efforts have moved towards developing novel 5‐HT_2C_R ligands that target topographically distinct allosteric 5‐HT_2C_R site(s). These distinct binding sites are less conserved than the orthosteric site, which is proposed to improve safety profiles with a reduced likelihood of on/off target side effects [12, 13]. This new era of drug discovery has resulted in the development of several selective 5‐HT_2C_R positive allosteric modulators (PAMs; [14, 15, 16, 17]).

In this study, we aimed to examine the effects of emerging 5‐HT_2C_R‐based PAMs on alcohol binge drinking and alcohol‐seeking in mice. To do this, we tested the acute effects of pretreatment doses of lorcaserin, CYD‐1‐79 [16], VA012 [15] and CTW0415 [17] on alcohol binge drinking in a drinking‐in‐the‐dark paradigm. Lorcaserin reduced alcohol consumption at all doses tested, yet 5‐HT_2C_R positive allosteric modulation at the doses tested did not reduce alcohol binge drinking. Following on from this, and based on our human data showing that lorcaserin reduced alcohol craving [10], we tested all compounds in a model of alcohol seeking. We used the context‐induced reinstatement following punishment‐imposed abstinence model ([18, 19], with slight modifications) and found that lorcaserin reduced alcohol‐seeking behaviour; however, 5‐HT_2C_R PAMs at the doses tested did not alter alcohol‐seeking behaviour. These data suggest that 5‐HT_2C_R PAMs may lack intrinsic efficacy for modulating alcohol use. However, future investigations of 5‐HT_2C_R PAMs in combination with a low‐dose orthosteric 5‐HT_2C_ agonist may be useful to further probe their therapeutic potential against aberrant alcohol use.

## 2 Materials and Methods

### 2.1 Ethics Statement and Reagents

All experiments were performed in accordance with the National Health and Medical Research Council (NHMRC) Australian Code for the Care and Use of Animals for Scientific Purposes (2013) and approved by The University of Newcastle Animal Care and Ethics Committee. Lorcaserin was obtained from Cayman Chemicals (Item 15521, Batch 0511311‐6) and VA012 was purchased from Axon Medchem (Axon 2889/Batch 1). CYD‐1‐79 and CTW0415 were synthesised by Dr. Zhou's laboratory at the University of Texas Medical Branch (UTMB) following the patents [20, 21] and reported protocols of UTMB [16, 17]. The compounds were validated by NMR and HPLC analysis (purity > 99%) and then delivered to Professor Lawrence and Dr. Campbell according to the mutually signed material transfer agreement (MTA) for further biological testing, prior to the approval by the Office of Technology Transfer (OTT) of the UTMB.

### 2.2 Animals

A total of 110 female C57BL/6JAusB mice (10–12 weeks old) were obtained from The Australian Resource Centre, Perth, Western Australia or Australian BioResources, New South Wales. Eight mice were excluded because their alcohol consumption was low throughout Context A self‐administration training (< 0.3 g/kg/session). Mice in binge drinking studies (Experiment 1) were individually housed. Mice in self‐administration studies (Experiment 2) were group housed during experiments (2–5 mice per cage) with standard bedding and enrichment. Food (Gordon's Speciality Feed) and water were available *ad libitum*, and all mice were maintained on a reverse 12‐h light/dark cycle (0700 lights off).

### 2.3 Experiment 1: Binge Drinking Following Acute 5‐HT_2C_R Modulation

**FIGURE 1:**
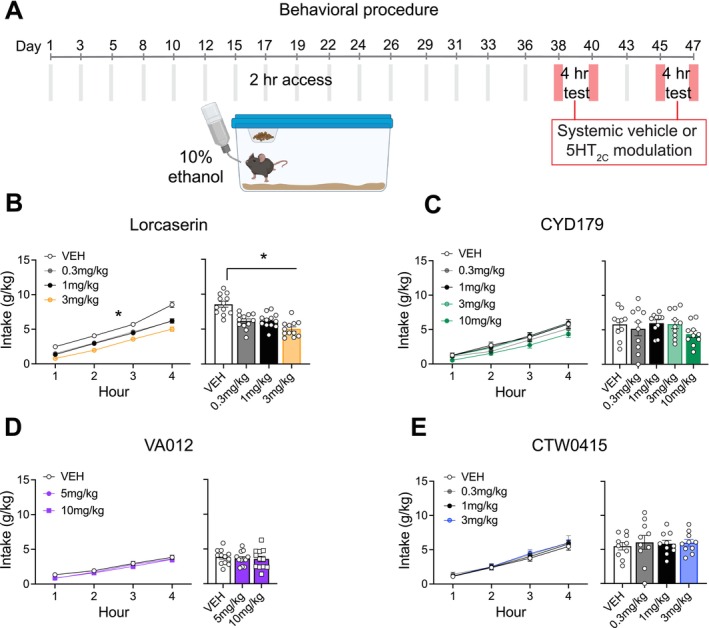
Experiment 1: Binge drinking following acute 5‐HT_2C_ modulation. (A) Outline of the behavioural procedure for Experiment 1. Created with BioRender.com. (B) Lorcaserin reduced binge alcohol consumption (g/kg) at all doses tested. (C) CYD‐1‐79 did not change binge alcohol consumption (g/kg) at any dose tested. (D) VA012 administration did not change binge alcohol consumption (g/kg) at any dose tested. (E) CTW0415 administration did not change binge alcohol consumption (g/kg) at any dose tested. Data presented as mean ± standard error mean. *p < 0.05.

A total of 53 female mice were used in this study. The drinking‐in‐the‐dark procedure was adapted from previously published work (Figure 1A) [22, 23, 24]. Here, water bottles were replaced with bottles of 10% v/v ethanol for 2 h beginning 3 h into the dark cycle (i.e., 10 am) [23, 25]. Alcohol was given to mice every Monday, Wednesday and Friday for 16 sessions. Prior to two of these sessions, mice received two vehicle habituation injections that corresponded to their drug group allocation (see below). Alcohol bottles were prepared in tap water from 100% (v/v) ethanol and weighed before and after the session to measure consumption (grams). Spillage was accounted for using a ‘spill’ bottle with 10% v/v ethanol in an empty cage during the session. Total alcohol consumption (grams per kilogram) was calculated for each session, using the weight difference between the beginning and end of the session, minus spillage, multiplied by 0.986 (density of 10% ethanol) and divided by the weight of the mouse in the cage.

#### 2.3.1 Binge Drinking Test

Please refer to Table 1 for the 5‐HT_2C_R modulators delivered at test, the number of mice in each test group, the pre‐treatment time, the corresponding vehicle solution, the dose of compound delivered and the route of administration. All compounds were delivered at 10 mL/kg. Using a within‐subjects, Latin‐square design, mice received vehicle and test compound injections prior to an extended ethanol binge drinking session (4 h access). Food (grams) and water (millilitres) consumption for each session was monitored at baseline, 6 h post‐compound administration and 24 h post‐compound administration. Mice received two injections during test weeks, with a minimum of 48 h between injections. The half‐life of lorcaserin, CYD‐1‐79, VA012 and CTW0415 is ~3 h, ~5 h, ~6 h and ~5 h respectively; no additional effects on behaviour using the within‐subjects test design were expected [12, 15, 17, 26]. A 2‐h binge drinking session was interweaved with test sessions on the days the drug was not administered. For the VA012 compound, two cohorts of mice were tested. Cohort 1 was tested at doses 0 mg/kg, 0.3 mg/kg, 1 mg/kg and 3 mg/kg; cohort 2 was tested at doses 0 mg/kg, 5 mg/kg and 10 mg/kg.

**TABLE 1:** Binge drinking test methods. Methods of 5‐HT_2C_R modulation for test. s.c., subcutaneous; i.p., intraperitoneal; DMSO, dimethyl sulfoxide.

5‐HT_2C_R modulator	Number of mice	Pre‐treatment time	Vehicle	Doses tested (mg/kg)	Route of administration
Lorcaserin	12	20 min	0.9% sterile saline	0.3; 1; 3	s.c
CYD‐1‐79	10	15 min	0.9% sterile saline	0.3; 1; 3; 10	i.p
VA012	Cohort 1 (9) Cohort 2 (12)	30 min	Acidified 3% DMSO in 0.9% sterile saline	0.3; 1; 3; 5; 10	s.c
CTW0415	10	30 min	10% DMSO and 90% 2‐hydroxypropyl‐beta‐cyclodextrin	0.3; 1; 3	i.p

### 2.4 Experiment 2: Alcohol‐Seeking Following Acute 5‐HT_2C_R Modulation

A total of 49 female mice were used in this study. The behavioural procedure was the same as recently published [24].

### 2.5 Apparatus

Standard operant chambers (Med Associates) enclosed in sound‐attenuating chambers were used for alcohol self‐administration. Each chamber was equipped with two retractable levers on either side of a central liquid receptacle. An active lever press resulted in the delivery of 10% v/v ethanol (13 μL/delivery) into the receptacle delivered via a 19‐gauge needle connected to a 3 mL syringe, controlled by a PHM‐200 syringe pump (Med Associates). An inactive lever press had no programmed consequence. Grid floors were connected to shockers (ENV‐414, Med Associates). Contexts A and B were manipulated in a similar way to our previous studies [18, 24]: background (stripes/none), illumination level (cue/no cue light), olfaction (vanilla essence/none), background noise (white noise off/on). The context used during either self‐administration or punishment was counterbalanced across mice, and no effects of context on alcohol consumption at baseline were observed.

### 2.6 Behavioural Procedure (Four Phases)

#### 2.6.1 Phase 1: Binge Drinking

The drinking‐in‐the‐dark procedure was identical to Experiment 1, where mice were exposed to 10% v/v ethanol in their home cage for 2 h. Alcohol was given to mice from 10 weeks of age on alternating days of the week for six sessions.

#### 2.6.2 Phase 2: Context A Self‐Administration Training

Mice underwent daily (weekday) operant sessions for 30 min, under a fixed‐ratio (FR‐1) schedule of reinforcement for 15 sessions. During this phase, one active lever press resulted in the delivery of 13 μL of 10% v/v ethanol paired with a 2‐s tone‐cue. This was followed by a 3‐s timeout period where lever presses were recorded but not reinforced. To facilitate operant acquisition, the inactive lever was absent during the first six sessions and introduced at session seven. Prior to two Context A training sessions, mice received habituation injections. For accurate alcohol consumption measurements at the end of the operant session, residual solution in the central receptacle was withdrawn with a 1 mL syringe and recorded. Context A training was conducted between 0900 and 1030 h.

#### 2.6.3 Phase 3: Context B Punishment

Following Context A self‐administration training, mice were trained to self‐administer alcohol in an alternate context (Context B) under the same FR‐1 administration schedule described above. Here, every second reinforced active lever press resulted in the delivery of a 0.5 s foot shock. Thus, punished active lever responses resulted in a foot shock, 2 s house light illumination, alcohol delivery and 2 s tone. Inactive lever presses had no programmed consequence. Mice were punished in Context B for at least 3 days, with a systemic habituation injection occurring prior to the second day in Context B. Day 1 in Context B had no delivery of foot shock and served as an acclimatisation day to the change in environment. The following day, every second lever press resulted in the delivery of 0.2 mA foot shock. The third punishment day resulted in the delivery of 0.3 mA foot shock. An extra punishment day was conducted at 0.3 mA for any mice that had greater than 50 active lever presses on Day 2.

#### 2.6.4 Phase 4: Alcohol‐Seeking

Mice underwent 2 days of alcohol‐seeking tests the day after Context B punishment‐imposed voluntary abstinence. Here, mice were examined for alcohol‐seeking behaviour in both Context A and Context B. The order of testing of the two contexts was counterbalanced. The drug type assigned to each mouse remained the same for the two test days. Alcohol‐seeking tests (relapse test) followed an FR‐1 schedule of reinforcement, where an active lever press resulted in the delivery of the 2 s tone; however, no alcohol or foot shock was delivered.

On test days, mice were randomly assigned to either the vehicle (0.9% sterile saline, s.c.) or lorcaserin (1 mg/kg, s.c.) and given injections 20 min prior to the alcohol‐seeking test. The dose of lorcaserin chosen for the alcohol‐seeking test was based on effects observed during binge drinking sessions and expected effects on food consumption, along with previous research [27].

A second group of mice was randomly assigned either vehicle (0.9% sterile saline, i.p.) or CYD‐1‐79 (3 mg/kg, i.p.) and given injections 15 min prior to the alcohol‐seeking test. The dose of CYD‐1‐79 chosen for the alcohol‐seeking test was based on previous research demonstrating that higher doses impacted spontaneous locomotor activity [16].

A third group of mice was randomly assigned either vehicle (acidified 3% DMSO in 0.9% sterile saline, s.c) or VA012 (5 mg/kg, s.c) and given injections 30 min prior to the alcohol‐seeking test. The dose of VA012 chosen for the alcohol‐seeking test was based on our pilot data demonstrating no effect on food and water consumption at this dose. To reduce the number of mice used in these studies, all mice in this experimental group were re‐trained in Context A for four sessions and re‐punished in Context B for two sessions. This was then followed by a second alcohol‐seeking test in both Context A and Context B with either acute VA012 or vehicle administration (the opposite drug to what mice received in the first test). We have previously shown that test context order does not influence alcohol‐seeking behaviour on re‐test [24]. Additionally, Test Context Order was included as a covariate in these analyses.

### 2.7 Statistical Analysis

Statistical analyses were conducted using JASP V16.2 and graphs were created with Graphpad Prism 10.50. Ethanol consumption for Experiment 1 was assessed as grams of ethanol consumed per kilogram of body weight. A repeated measures analysis of variance (ANOVA) assessed the effect of Dose and Timepoint on ethanol consumption. Food and water consumption were assessed using a repeated measures ANOVA with Dose and Timepoint as the within‐subject factors. For Experiment 2, data were analysed separately for the four behavioural phases: binge drinking, Context A self‐administration, Context B punishment and the alcohol‐seeking tests. Binge drinking, self‐administration and punishment data were analysed using a repeated measures analysis of variance (ANOVA) examining a within‐subjects effect of Day. ANOVA was used to analyse the alcohol‐seeking tests; the within‐subjects factor was Context (Context A, Context B) and the between‐subjects factor was Drug (Vehicle and lorcaserin or CYD‐1‐79). For the VA012 alcohol‐seeking test cohort, there were two within‐subject factors (Context and Drug), and Test Context Order was included as a covariate in analyses. The percentage change from baseline was calculated as ((alcohol‐seeking test active lever presses) − (average active lever presses of the last five Context A sessions)) / (average active lever presses of the last five Context A sessions) * 100. Significant interaction effects (*p* < 0.05) were followed up with Tukey post hoc comparisons. Investigators who performed drug administration and analyses were blinded to group assignments.

## 3 Results

### 3.1 Experiment 1: Binge Drinking Following Acute 5‐HT_2C_R Modulation

Figure 1A depicts the timeline of testing for the distinct drugs during the binge drinking experiment.

#### 3.1.1 Binge Drinking Following Acute Lorcaserin Administration

**FIGURE 2:**
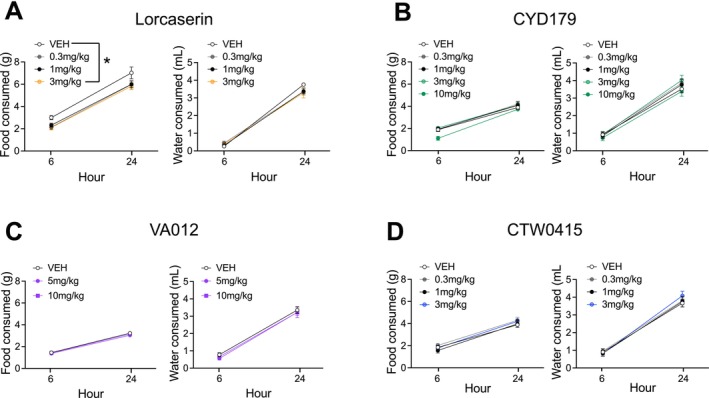
Effect of acute 5‐HT_2C_ modulation on food and water intake. (A) Lorcaserin (3 mg/kg) reduced food intake compared to vehicle controls (left). Lorcaserin did not influence water intake (right). (B) CYD‐1‐79 did not influence food or water intake at the doses tested. (C) VA012 did not influence food or water intake at any dose tested. (D) CTW0415 did not influence food or water intake at any dose tested. Data presented as mean ± standard error mean. *p < 0.05.

During binge drinking test sessions, a main effect of lorcaserin dose was reported (*F*_3,33_ = 28.678, *p* < 0.001) and a dose‐dependent effect on the timepoints measured (Figure 1B; Dose × Timepoint interaction *F*_9, 99_ = 7.956, *p* < 0.001). Post hoc tests showed that lorcaserin reduced alcohol consumption at each dose tested compared to vehicle (*p*'s < 0.05). There was also an effect of lorcaserin dose on food consumption (Figure 2A; main effect of Dose *F*_3, 33_ = 3.497, *p* = 0.026) but no Dose × Timepoint interaction (*F*_3,33_ = 0.437, *p* = 0.728). Post hoc tests showed that this was mostly driven by a reduction in food consumption at the highest dose of lorcaserin tested (3 mg/kg; *p* < 0.05). There was no significant effect of lorcaserin dose on water consumption at the tested timepoints (Figure 2A; Dose *F*_3,33_ = 0.350, *p* = 0.789; Dose × Timepoint interaction *F*_3, 33_ = 2.343, *p* = 0.091).

#### 3.1.2 Binge Drinking Following Acute CYD‐1‐79 (5‐HT_2C_R PAM) Administration

There was no significant effect of CYD‐1‐79 dose on grams per kilogram ethanol consumed during the binge drinking test (Figure 1C; main effect of Dose *F*_4, 36_ = 1.471, *p* = 0.231). There was a trend towards a significant effect of CYD‐1‐79 dose on food consumption (Figure 2B; main effect of Dose *F*_4, 36_ = 2.472, *p* = 0.062). Post hoc tests revealed that this trend was mostly driven by a reduction in food consumption at the 10 mg/kg dose, 6 h post‐CYD‐1‐79 administration (*p* = 0.03). There was no significant effect of CYD‐1‐79 dose on water consumption (Figure 2B; main effect of Dose *F*_4, 36_ = 1.043, *p* = 0.399).

#### 3.1.3 Binge Drinking Following Acute VA012 (5‐HT_2C_R PAM) Administration

Cohort 1 tested the effect of VA012 at doses up to 3 mg/kg on binge alcohol consumption. There was no effect of doses up to 3 mg/kg of VA012 on grams per kilogram of alcohol consumed (Figure S1A; main effect of Dose *F*_3, 24_ = 1.257, *p* = 0.311). Cohort 2 tested the effect of VA012 at doses of 5 and 10 mg/kg on binge alcohol consumption. There was no effect of any dose of VA012 tested on grams per kilogram of alcohol consumed (Figure 1D; main effect of Dose *F*_2, 22_ = 0.950, *p* = 0.402). For cohort 1, there was also no effect of VA012 at doses tested (up to 3 mg/kg) on food (Figure S1B; main effect of Dose *F*_3, 24_ = 1.006, *p* = 0.407) or water consumption (Figure S1B; main effect of Dose *F*_3, 24_ = 0.504, *p* = 0.683). For cohort 2, there was no effect of VA012 at doses tested (5 and 10 mg/kg) on food (main effect of Dose *F*_2, 22_ = 1.827, *p* = 0.184) or water consumption (main effect of Dose *F*_2, 22_ = 0.452, *p* = 0.642) (Figure 2C).

#### 3.1.4 Binge Drinking Following Acute CTW0415 (5‐HT_2C_R PAM) Administration

There was no significant effect of any dose of CTW0415 tested on grams per kilogram alcohol consumed during binge drinking (Figure 1E; main effect of Dose *F*_3, 27_ = 0.180, *p* = 0.909). There was also no effect of CTW0415 at doses tested on food consumption (Figure 2D; main effect of Dose *F*_3, 27_ = 0.948, *p* = 0.431) or water consumption (Figure 2D; main effect of Dose *F*_3, 27_ = 0.191, *p* = 0.902).

### 3.2 Experiment 2: Alcohol‐Seeking Following Acute 5‐HT_2C_ Modulation

**FIGURE 3:**
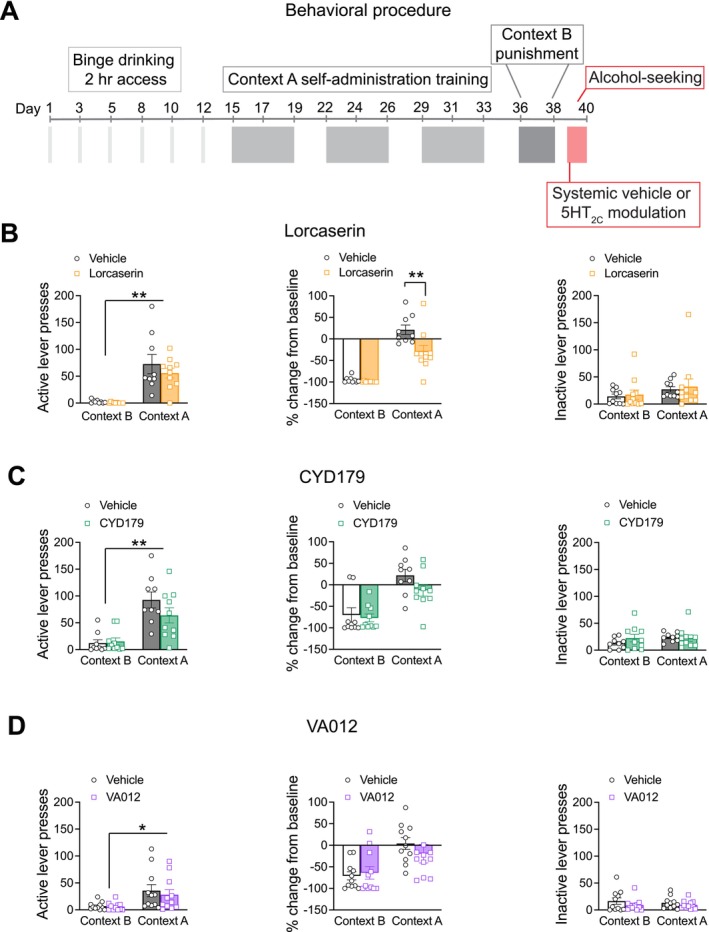
Experiment 2: Alcohol‐seeking following acute 5‐HT_2C_ modulation. (A) Outline of the behavioural procedure for Experiment 2. (B) Mice increased alcohol‐seeking behaviour in Context A versus Context B (left). Lorcaserin reduced alcohol‐seeking behaviour in Context A when accounting for baseline responding (middle). There was no effect of lorcaserin on inactive lever presses during the alcohol‐seeking test (right). (C) Mice had increased active lever responding in Context A versus Context B during the alcohol‐seeking test (left). There was no effect of CYD‐1‐79 on alcohol‐seeking behaviour (middle) or inactive lever pressing during the test (right). (D) Mice increased alcohol‐seeking behaviour in Context A compared to Context B (left). VA012 had no effect on alcohol‐seeking when accounting for baseline (middle) or inactive lever responding (right). Data presented as mean ± standard error mean. *p < 0.05; **p < 0.01.

Figure 3A depicts the timeline of testing for the distinct drugs during the alcohol‐seeking experiment.

#### 3.2.1 Alcohol‐Seeking Following Acute Lorcaserin Administration

Mice consumed high quantities of alcohol during the binge‐drinking phase of the behavioural procedure (~2 g/kg over 2 h; data not shown). During Context A self‐administration, mice increased their active lever responding for alcohol across days (Figure S2A; main effect of Day *F*_14, 252_ = 8.317, *p* < 0.001) and had no change in inactive lever presses across days (Figure S2A; main effect of Day *F*_6, 108_ = 1.196, *p* = 0.314). For Context B punishment, mice reduced active lever responding for alcohol with increasing foot shock intensity (from 0.2 mA on Day 2 to 0.3 mA on Day 3) (Figure S2A; main effect of Day *F*_2, 36_ = 137.074, *p* < 0.001). Inactive lever responding in Context B also reduced across days (Figure S2A; main effect of Day *F*_2, 36_ = 10.131, *p* < 0.001).

For the alcohol‐seeking test, mice pressed the active lever more during Context A versus Context B (Figure 3B; main effect of Context *F*_1,17_ = 42.354, *p* < 0.01). Lorcaserin (1 mg/kg) did not influence the number of active lever presses during the alcohol‐seeking reinstatement test (Context × Drug interaction *F*_1,17_ = 0.575, *p* = 0.459). To account for individual differences observed in lever pressing, the percentage change from baseline was calculated. Here, lorcaserin reduced lever responding in Context A when compared to vehicle controls (Figure 3B; Context × Drug interaction *F*_1,17_ = 6.199, *p* = 0.023; post hoc *p* < 0.01), without influencing the number of inactive lever presses during the alcohol‐seeking test (Figure 3B; Context × Drug interaction *F*_1,17_ = 0.004, *p* = 0.948).

#### 3.2.2 Alcohol‐Seeking Following Acute CYD‐1‐79 Administration

Mice consumed high quantities of alcohol during the binge drinking phase of the behavioural procedure (2 g/kg over 2 h; data not shown). During Context A self‐administration training, there was an effect of Day on active lever responding for alcohol (Figure S2B; main effect of Day *F*_14, 252_ = 3.361, *p* < 0.001) with post hoc tests showing changes in active lever responding on Days 3, 7 and 9 of training (*p*'s < 0.05). There were also changes in inactive lever presses across day (Figure S2B; main effect of Day *F*_6, 108_ = 4.107, *p* < 0.001). Post hoc tests showed that these changes in inactive lever presses were driven by greater inactive responding on Days 9 and 10 of training (*p*'s < 0.05). For Context B punishment, mice reduced active lever responding for alcohol with increasing foot shock intensity (Figure S2B; main effect of Day *F*_2, 36_ = 34.511, *p* < 0.001). Inactive lever responding in Context B also reduced across day (Figure S2B; main effect of Day *F*_2, 36_ = 8.969, *p* < 0.001).

For the alcohol‐seeking test following acute CYD‐1‐79 administration, mice pressed the active lever more during Context A versus Context B (Figure 3C; main effect of Context *F*_1,17_ = 37.271, *p* < 0.01), yet CYD‐1‐79 did not influence the number of active lever presses during the alcohol‐seeking reinstatement test (Context × Drug interaction *F*_1,17_ = 2.299, *p* = 0.148). CYD‐1‐79 also did not change alcohol‐seeking behaviour when baseline lever responding was accounted for (Figure 3C; Context × Drug interaction *F*_1,17_ = 1.719, *p* = 0.207), and did not influence the number of inactive lever presses during the alcohol‐seeking test (Figure 3C; Context × Drug interaction *F*_1,17_ = 3.776, *p* = 0.069).

#### 3.2.3 Alcohol‐Seeking Following Acute VA012 Administration

Mice consumed high quantities of alcohol during the binge drinking phase of the behavioural procedure (2.1 g/kg over 2 h; data not shown). During Context A self‐administration, there was an effect of Day on active lever responding for alcohol (Figure S2C; main effect of Day *F*_14, 140_ = 2.310, *p* = 0.007) with post hoc tests showing changes in active lever responding on Day 3 of training (*p*'s < 0.05). There were reductions in inactive lever presses across days during Context A training (Figure S2C; main effect of Day *F*_6, 60_ = 6.875, *p* < 0.001). During Context B punishment, mice reduced active lever responding for alcohol with increasing foot shock intensity (Figure S2C; main effect of Day *F*_2, 20_ = 43.190, *p* < 0.001). There were no changes in inactive lever responding in Context B (Figure S2C; main effect of Day *F*_2, 20_ = 1.506, *p* = 0.246).

For the alcohol‐seeking test following acute VA012 administration, mice pressed the active lever more during Context A versus Context B (Figure 3D; main effect of Context *F*_1,10_ = 6.453, *p* = 0.029). VA012 did not influence the number of active lever presses during the alcohol‐seeking reinstatement test (Context × Drug interaction *F*_1,10_ = 0.509, *p* = 0.492). Importantly, Test Context Order was not a significant covariate in the final alcohol‐seeking test and had no significant influence on active lever presses (*p* > 0.05). VA012 also did not change alcohol‐seeking behaviour when baseline lever responding was accounted for (Figure 3D; Context × Drug interaction *F*_1,10_ = 0.704, *p* = 0.421). VA012 also did not influence the number of inactive lever presses during the alcohol‐seeking test (Figure 3D; Context × Drug interaction *F*_1,10_ = 2.204, *p* = 0.169).

## 4 Discussion

The 5‐HT_2C_R has been implicated in appetitive and motivated behaviours, and 5‐HT_2C_R agonists can suppress food intake [28, 29], drug intake [30, 31] and alcohol consumption [10]. In the context of AUD, preclinical literature suggests that enhancing serotonin function can decrease alcohol use‐associated behaviours in rodent models. Alcohol‐preferring rats have low serotonin levels, which contribute to their alcohol seeking and drinking phenotypes [32]. Ro60‐0175, a 5‐HT_2C_R agonist, reduced alcohol self‐administration in male rats [7]. SB 206,553 (1 mg/kg, i.p.), a 5‐HT_2C_R antagonist, blocked mCPP (a 5‐HT_2C/1B_R agonist) induced generalisation to an ethanol cue in a drug discrimination paradigm [33]. Further, lorcaserin reduced alcohol binge drinking in male mice and in female alcohol‐preferring P rats in a two‐bottle choice paradigm following both acute and chronic administration [27, 34]. In contrast, antagonising this receptor with SB‐242084 increased alcohol intake in male rats [7]. These data demonstrate that 5‐HT_2C_R signalling is associated with distinct processes that contribute to alcohol intake.

Here, we showed that full 5‐HT_2C_R agonism with lorcaserin, at all tested doses, reduced alcohol binge drinking in a drinking‐in‐the‐dark paradigm in female mice. We also investigated the role of lorcaserin in a behavioural paradigm of alcohol‐seeking. Our results showed that mice learned to self‐administer alcohol through active lever presses during Context A training. Once moved to Context B (active lever now paired with punishment), mice pressed the active lever significantly less once foot shock was delivered (Days 2 and 3), extinguishing alcohol‐seeking behaviour. Importantly, all mice reinstated alcohol‐seeking behaviour during the relapse test in Context A, the alcohol‐associated context, but not when tested in Context B, the punishment context, agreeing with previous literature in rats [18, 35, 36] and mice [24]. In our model of context‐induced reinstatement following punishment‐imposed abstinence, lorcaserin was also effective, although to a lesser extent, in reducing alcohol‐seeking when compared to baseline levels of consumption, agreeing with previous literature on the effect of 5‐HT_2C_R agonism in reducing two distinct core features associated with AUD [34, 37]. These are the first findings, to the best of our knowledge, demonstrating an effect of lorcaserin on reducing alcohol‐seeking following punishment‐imposed abstinence. Of note, the highest dose of lorcaserin (3 mg/kg) led to a sustained reduction in food intake over a period of 24 h posttreatment, but no changes in water intake were observed, not surprising given that lorcaserin (Belviq) was previously approved as an appetite suppressant for the treatment of obesity [38].

Considering that Belviq was withdrawn from the market due to concerns over potential off‐target effects associated with long‐term use [11], we tested 5‐HT_2C_R PAMs to investigate if similar findings could be observed through targeting allosteric sites. We tested VA012, CYD‐1‐79, both PAMs that enhance serotonin efficacy with no significant off‐target binding, and CTW0415, a compound with improved pharmacological and physiochemical properties when compared to older PAMs [15, 16, 17]. Although preclinical behavioural studies are limited using these compounds, CYD‐1‐79 has been tested in the context of substance use disorders (SUDs) and shown to reduce spontaneous ambulatory activity and attenuate cocaine‐induced cue reactivity in rats [16]. Nonetheless, we failed to observe an effect of any of the tested PAMs in reducing alcohol binge drinking. In the relapse model, likewise, none of the tested PAMs were able to reduce alcohol seeking following punishment‐imposed abstinence. Further, none of the PAMs impacted food or water intake. This supports the lack of potential side effects associated with 5‐HT_2C_R PAMs versus lorcaserin, the orthosteric agonist. Given the novelty of these compounds, there is minimal existing research on potential side effects. However, low doses (2 mg/kg) of VA012 have been previously shown to reduce food intake in food‐restricted rats [15]. In our study, mice had free access to food prior to the test. These data suggest that the metabolic state of the animal is an important consideration when examining the impact of 5‐HT_2C_R modulation and associated side effects [39].

One point worth considering is whether the 5‐HT_2C_R PAMs used in the current study were active, particularly given there was no effect on food and water intake. In our study, the highest dose of CYD‐1‐79 had a near‐significant effect on food intake at 6 h post‐administration. Additionally, we have pilot data in male mice (not shown) demonstrating that 1 and 3 mg/kg of VA012 increased water intake. While we did not directly test the active state of these compounds, future studies using electrophysiological recordings will be required to completely understand the effect of these 5‐HT_2C_R PAMs on cell function.

A possible explanation for the observed actions of lorcaserin in reducing alcohol intake but the lack of effect of PAMs could be due to the PAMs' requirements for the endogenous agonist (5‐HT) or the 5‐HT_2C_R agonist lorcaserin to activate the 5‐HT_2C_R directly [13, 16, 40]. Dynamic alterations in 5‐HT_2C_R expression and 5‐HT levels have been reported in which intermittent alcohol binge drinking and chronic intake could lead to a reduction in circulating serotonin levels in the brains of rodents and humans, and either a decrease or increase in receptor expression and function depending on the brain region [41, 42, 43], potentially contributing to the lack of PAM efficacy. Abstinence from ethanol following prolonged exposure in a two‐bottle choice paradigm leads to a reduction in tryptophan hydroxylase‐2 enzyme expression and increased 5‐HT_2C_R editing efficiency in the hippocampus of mice [44]. Similarly, chronic ethanol vapour exposure contributes to increased expression of 5‐HT_2C_R RNA editing in the nucleus accumbens [45], mediated via adenosine deaminase acting on RNA‐2 enzyme [46]. Of note, 5‐HT_2C_R undergoes pre‐RNA editing and this receptor can function in 32 distinct mRNA isoforms and encode up to 24 different receptor protein isoforms in rodents and humans [47, 48]. Hence, increased editing may contribute to decreased receptor function, which can directly impact PAM efficacy [49]. Lastly, some of the behavioural effects observed following lorcaserin administration could be attributed to the recruitment of downstream signalling cascades and/or biased signalling compared to the PAMs tested here [50, 51, 52].

A potential avenue worth exploring is dual targeting of 5‐HT_2C_R distinct binding sites with a full agonist and PAM, or alternatively bitopic ligands that act as AGO‐PAMs. As an example, a low dose of lorcaserin in combination with a PAM may enhance the efficacy of lorcaserin at even lower doses with potentially improved safety and tolerability, circumventing previous side‐effect concerns. Although no published study has tested this combination with regard to alcohol use, lorcaserin has been used in combination previously to reduce binge intake of ethanol and food and reduce nicotine self‐administration. For example, combination of low dose lorcaserin (1 mg/kg) with pimavanserin (0.3 mg/kg; a 5‐HT_2A_R antagonist/inverse agonist) reduced food binge episode frequency and intake and weight gain associated with high fat food exposure in male rats [53]. Further, combining low doses of lorcaserin (0.3 and 0.6 mg/kg) with varenicline (1 mg/kg; Chantix) reduced nicotine self‐administration and reinstatement in male rats to a greater extent than either drug alone, without impacting food intake [54]. Lastly, in a model of alcohol binge drinking in mice, combination of low doses of lorcaserin (0.375 and 0.75 mg/kg) with naltrexone (1 mg/kg) attenuated ethanol drinking over the 4‐h test [27], similar to our results. These data support 5‐HT_2C_R agonists for reduction of motivated behaviours across species and further investigation of combinations with PAMs to minimise side effect profiles.

Here, we showed that lorcaserin was effective in reducing binge drinking in mice as well as reducing relapse‐like behaviour within subjects. To the best of our knowledge, this is the first time 5‐HT_2C_R agonists have been tested using this relapse paradigm in mice. In contrast, the same effects were not observed with the 5‐HT_2C_R PAMs tested here. In addition, we have demonstrated the reproducibility of this model of relapse following punishment‐imposed abstinence of alcohol seeking. Despite not observing a main effect of VA012, CYD‐1‐79 and CTW0415 in reducing alcohol binge drinking and relapse to alcohol seeking in the paradigms used here, our lorcaserin data in combination with previous literature support further studies investigating the targeting of the 5‐HT_2C_ receptor in AUD research.

## Author Contributions

**Erin J. Campbell** and **Andrew J. Lawrence** designed the study; **Erin J. Campbell** and **Linh Tran** conducted all experiments; **Roberta G. Anversa** and **Erin J. Campbell** conducted analyses; **Andrew A. Bolinger**, **Kathryn A. Cunningham** and **Jia Zhou** provided 5‐HT_2C_R positive allosteric modulators and wrote/reviewed/edited this manuscript with all authors.

## Disclosure

This work was supported by an Australian Research Council Discovery Early Career Researcher Award (DE230100401), a NARSAD Young Investigator Grant from the Brain & Behaviour Foundation (32829) and a Hunter Medical Research Institute and University of Newcastle Brain Neuromodulation Seed Funding Grant to EJC. It was also supported by grants R21 MH093844 (JZ/KAC), R01 DA038446 (JZ/KAC), and P30 DA028821 (KAC) from the National Institutes of Health, the John D. Stobo, M.D. Distinguished Chair Endowment Fund (JZ), the Edith & Robert Zinn Chair in Drug Discovery Endowment Fund (JZ), the Chauncey Leake Distinguished Professor Endowment (KAC), and the Center for Addiction Sciences and Therapeutics at UTMB. AJL is supported by an NHMRC Synergy Grant (2009851). For open access, the author has applied a CC BY public copyright licence to any Author Accepted Manuscript version arising from this submission. We acknowledge support from the Victorian State Government Operational Infrastructure Scheme.

## Conflicts of Interest

KAC is a consultant for Delix Therapeutics unrelated to this study. All other authors declare no conflicts of interest, financial or otherwise.

## Supporting information


**Figure S1:** Binge drinking, food and water intake following low dose VA012 administration. (A) VA012 did not impact binge alcohol consumption at any dose tested (B) VA012 did not influence food or water intake at the doses tested. Data presented as mean ± standard error mean.

**Figure S2:** Alcohol self‐administration and punishment for each experimental cohort. (A) Context A active lever presses increased across training day in the lorcaserin experimental group (left). Context B active and inactive lever responding reduced across subsequent punishment days (right). The foot shock punishment ranged from 0 mA on Day 1, to 0.2 mA on Day 2 and 0.3 mA on Day 3. (B) There was variability in active and inactive lever pressing across Context A training for the CYD‐1‐79 cohort (left). Context B active and inactive lever presses reduced across training day (right). The foot shock punishment ranged from 0 mA on Day 1, to 0.2 mA on Day 2 and 0.3 mA on Day 3. (C) There was variability in active lever pressing across Context A training for the VA012 cohort (left). Context A inactive lever pressing reduced across training day (left). Context B active and inactive lever presses reduced across training day (right). The foot shock punishment ranged from 0 mA on Day 1, to 0.2 mA on Day 2 and 0.3 mA on Day 3. Data presented as mean ± standard error mean. ***p < 0.001.

## Data Availability

The data that support the findings of this study are available from the corresponding author upon reasonable request.
