# Supplementary figures and images for: The Effect of Serotonin 5‐HT2C Receptor Modulation on Binge Drinking and Alcohol‐Seeking in Female Mice
Source: Addict Biol. 2025 Nov 10;30(11):e70099. doi: 10.1111/adb.70099 (PMC12602058; doi:10.1111/adb.70099)

Figure S1

Binge drinking, food and water intake following low dose VA012 administration

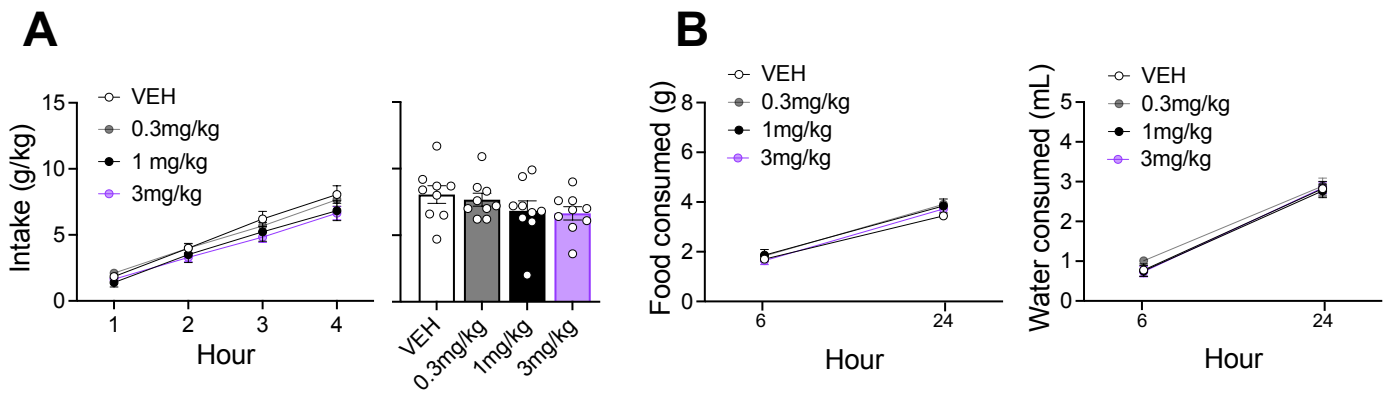

Supplement: Supplementary file 1 — Figure S1: Binge drinking, food and water intake following low dose VA012 administration. (A) VA012 did not impact binge alcohol consumption at any dose tested (B) VA012 did not influence food or water intake at the doses tested. Data presented as mean ± standard error mean. [file ADB-30-e70099-s001.pdf]

Figure S2

Alcohol self-administration and punishment for each experimental cohort

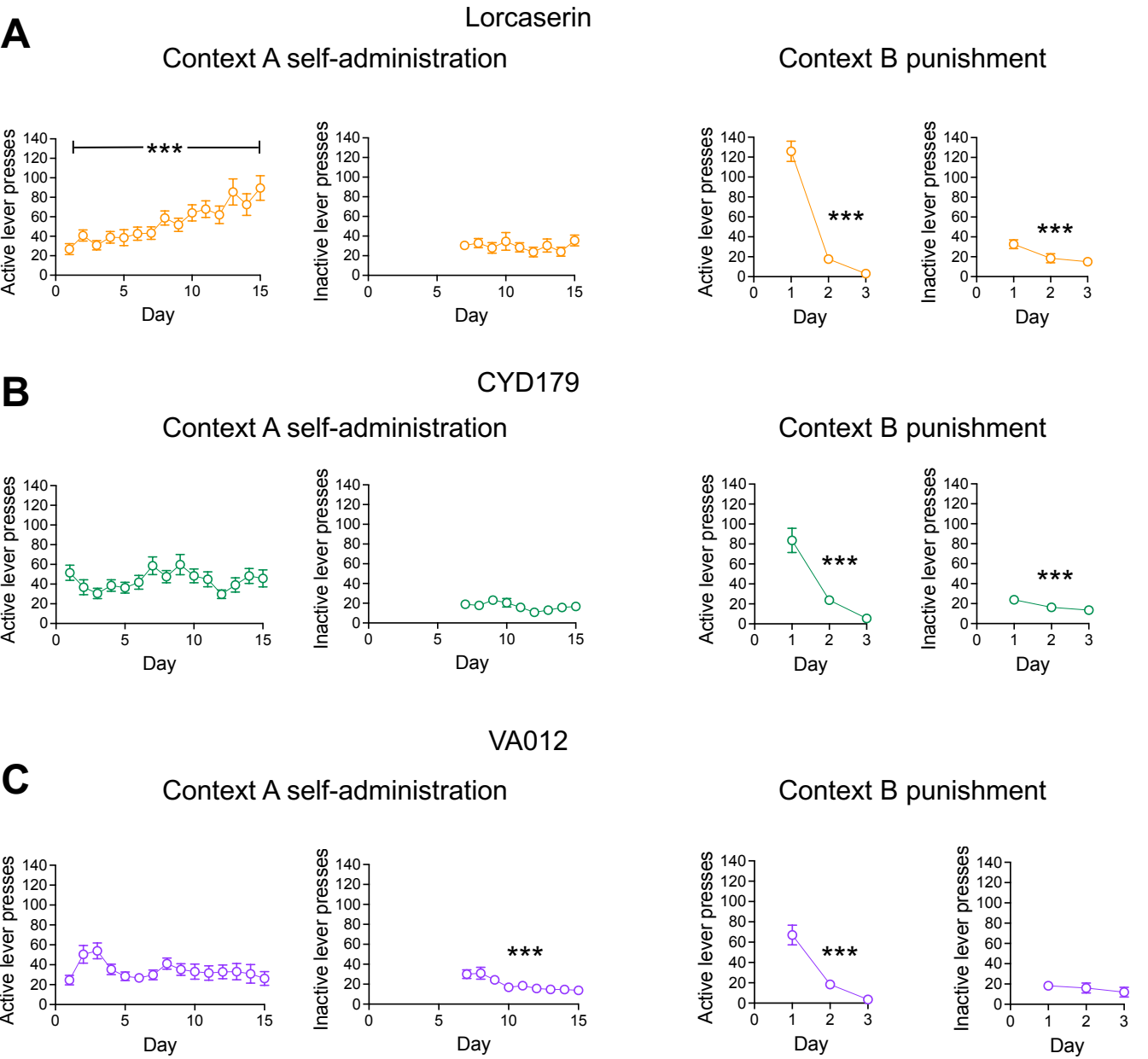

Supplement: Supplementary file 2 — Figure S2: Alcohol self‐administration and punishment for each experimental cohort. (A) Context A active lever presses increased across training day in the lorcaserin experimental group (left). Context B active and inactive lever responding reduced across subsequent punishment days (right). The foot shock punishment ranged from 0 mA on Day 1, to 0.2 mA on Day 2 and 0.3 mA on Day 3. (B) There was variability in active and inactive lever pressing across Context A training for the CYD‐1‐79 cohort (left). Context B active and inactive lever presses reduced across training day (right). The foot shock punishment ranged from 0 mA on Day 1, to 0.2 mA on Day 2 and 0.3 mA on Day 3. (C) There was variability in active lever pressing across Context A training for the VA012 cohort (left). Context A inactive lever pressing reduced across training day (left). Context B active and inactive lever presses reduced across training day (right). The foot shock punishment ranged from 0 mA on Day 1, to 0.2 mA on Day 2 and 0.3 mA on Day 3. Data presented as mean ± standard error mean. ***p < 0.001. [file ADB-30-e70099-s002.pdf]
